# Antiadhesive and Antibiofilm Effect of Malvidin-3-Glucoside and Malvidin-3-Glucoside/Neochlorogenic Acid Mixtures upon *Staphylococcus*

**DOI:** 10.3390/metabo12111062

**Published:** 2022-11-03

**Authors:** Sara Silva, Eduardo M. Costa, Manuela Machado, Rui Morais, Conceição Calhau, Manuela Pintado

**Affiliations:** 1 CBQF—Centro de Biotecnologia e Química Fina—Laboratório Associado, Escola Superior de Biotecnologia, Universidade Católica Portuguesa, Rua Diogo Botelho 1327, 4169-005 Porto, Portugal; 2 Nutrição e Metabolismo, NOVA Medical School, Universidade Nova de Lisboa, Campo dos Mártires da Pátria, 130, 1169-056 Lisboa, Portugal; 3 CINTESIS, Centro de Investigação em Tecnologias e Serviços de Saúde, Universidade do Porto, 4200-450 Porto, Portugal

**Keywords:** anthocyanin, neochlorogenic acid, *Staphylococcus*, antibiofilm, antimicrobial, antiadhesion

## Abstract

Several reports on the biological activity of anthocyanin-rich extracts have been made. However, despite the association of said activity with their anthocyanin content, to the best of our knowledge, there are no previous works regarding the antimicrobial, antibiofilm and/or antiadhesive properties of anthocyanins alone. Therefore, the present work aimed to determine the effects of malvidin-3-glucoside, a major component of a previously reported extract, and the impact of its association with neochlorogenic acid (the only non-anthocyanin phenolic present in said extract), upon several *Staphylococcus* strains with varying resistance profiles. Results show that, while malvidin-3-glucoside and malvidin-3-glucoside/neochlorogenic acid mixtures were unable to considerably inhibit bacterial growth after 24 h, they still possessed an interesting antibiofilm activity (with reductions of biofilm entrapped cells up to 2.5 log cycles, metabolic inhibition rates up to 81% and up to 51% of biomass inhibition). When considering the bacteria’s capacity to adhere to plain polystyrene surfaces, the inhibition ranges were considerably lower (21% maximum value). However, when considering polystyrene surfaces coated with plasmatic proteins this value was considerably higher (45% for adhesion in the presence of extract and 39% for adhesion after the surface was exposed to extract). Overall, the studied anthocyanins showed potential as future alternatives to traditional antimicrobials in adhesion and biofilm formation prevention.

## 1. Introduction

Anthocyanins are natural, water-soluble pigments that attracted the interest of the scientific community given their vast array of potential applications, from being used as food additives to serving as a base in the development of new photovoltaic energy sources [1,2,3]. From a biological standpoint, anthocyanins have been associated with several health-promoting properties such as having antioxidant, anti-inflammatory, antiproliferative and anticarcinogenic activity as well as positive effects on blood sugar levels and in the cardiovascular and neurologic systems [4,5,6]. One of the potential biological activities of anthocyanins includes antimicrobial activity. In the literature, several different reports may be found on the antimicrobial capacity of different types of anthocyanin-rich extracts against a considerably wide range of microorganisms and, more recently, even reports of anthocyanin-rich extracts’ capacity to inhibit biofilm formation have emerged, with studies showing inhibition of *Pseudomonas aeruginosa*, *Salmonella enterica* and *S. aureus* [7,8]. However, the available literature regarding the effect of individual anthocyanins is considerably scarce and focused on planktonic cells. In nature, bacteria are scarcely found in this state, rather they are most often present in biofilms, survival structures notorious for their high resistance to antimicrobial agents. Therefore, the literature available regarding the antimicrobial potential of pure anthocyanins is not only scarce but, to the best of our knowledge, disregarding one very important factor—the impact upon bacterial biofilms [9].

*Staphylococcus* are one of the most commonly found pathogenic agents and possess several inherent characteristics that help them be so, namely their ubiquitous presence in humans and their propensity to develop antimicrobial resistance [10,11]. *S. aureus*, one highly recognized pathogen, has long since been split into two different groups regarding their antibiotic resistance: methicillin-sensitive *S. aureus* (MSSA) or methicillin-resistant *S. aureus* (MRSA). Recently, a new resistant group has emerged, the vancomycin-resistant *S. aureus* (VRSA) [11,12]. Furthermore, *S. epidermidis*, usually regarded as a commensal microorganism of human skin, has also emerged as an important pathogen as it has acquired resistance to methicillin (methicillin-resistant *S. epidermidis*, MRSE) [13].

A previous work showed that an anthocyanin-rich blueberry extract (whose most abundant anthocyanins were malvidins, particularly malvidin-3-glucoside (M3Glu)) with neochlorogenic acid (NChA) was capable of significantly inhibit the growth, adhesion and biofilm formation of *S. aureus* [1]. Therefore, in an attempt to better understand the real effect of each compound and establish a compound–effect relationship, the present work aimed to determine the antimicrobial potential of M3Glu (alone and in the presence of NChA) against an array of staphylococci strains with clinical relevance (MSSA, MRSA, VRSA and MRSE).

## 2. Materials and Methods

### 2.1. Microorganisms

Several *Staphylococcus* strains were used in this work. Clinical isolates (CIs, from urine), capable of establishing biofilms, of a methicillin-sensitive (MSSA CI) and a methicillin-resistant (MRSA CI) *S. aureus* were kindly provided by CHTMAD—Hospital Centre of Trás-os-Montes e Alto Douro (through Ph.D. Maria José Alves). Additionally, three reference (R) strains of *S. aureus* and one of *S. epidermidis* were also considered: MSSA ATCC 25923 (MSSA R), MRSA CCUG 60578 (MRSA R), vancomycin-resistant *S. aureus* ATCC 700699 (VRSA) and methicillin-sensitive *S. epidermidis* ATCC 51625 (MRSE).

### 2.2. Test Solutions

M3Glu was acquired from Extrasynthese (Lyon, France) and NChA was acquired from Sigma (St. Louis, MO, USA). Four different test solutions were prepared, two using only M3Glu (500 and 250 µg mL^−1^) and two using a mixture of M3Glu and NChA: 500 µg mL^−1^ M3Glu with 100 µg mL^−1^ NChA (cM3Glu/NChA) and 250 µg mL^−1^ M3Glu with 50 µg mL^−1^ NChA (M3Glu/NChA). All solutions were prepared using sterile saline (0.5% (*w v*^−1^) NaCl) for the adhesion assays or tryptic soy broth (TSB, Biokar Diagnostics, Beauvais, France) (supplemented with 1% (*w v*^−1^) glucose (Sigma, St. Louis, MO, USA) for the antibiofilm assays) and sterilized using a 0.22 µm sterile filter (Millipore, Billerica, MA, USA).

### 2.3. Time Inhibition Curves

Time inhibition curves were drawn as described elsewhere [1,14]. Briefly, the test solutions (specified in Section 2.2) were inoculated at 1% (*v v*^−1^) with an overnight inoculum and transposed into a 96-well microtiter (Nunc, Darmstadt, Germany) and incubated at 37 °C. The optical density (OD) at 660 nm was assessed for a 24 h period, at 1 h intervals, using a microplate reader (Fluostar Optima; BMG Labtech, Ortenberg, Germany). A positive control was drawn using plain TSB without an antimicrobial agent and TSB was added as a negative control. Each condition was assayed in triplicate.

### 2.4. Total Planktonic Viable Cell Determination

Test solutions were inoculated as described in Section 2.3. After 24 h incubation, the total viable cells were determined using the drop method as described by Miles, Misra [15]. Briefly, decimal dilutions were plated in plate count agar (PCA, Biokar Diagnostics, Beauvais, France) and incubated at 37 °C for 24 h. Plain TSB was used as a positive control and each assay was performed in triplicate.

### 2.5. Antibiofilm Activity

The test solutions (specified in Section 2.2) were inoculated at 2% (*v v*^−1^) with an overnight inoculum, aliquots were distributed into 96-well microtiters (Nunc, Darmstadt, Germany) and incubated for 24 h at 37 °C. Afterwards, the content of each well was carefully discarded, washed (thrice using sterile phosphate-buffered saline pH 7.4 (PBS)) to remove non-adherent cells and then used to determine the biofilm-entrapped cells, biomass, metabolic activity and protein content. Plain TSB supplemented with 1% (*w v*^−1^) glucose was used as a positive control. Each assay was performed in triplicate.

#### 2.5.1. Biofilm-Entrapped Viable Cells

To quantify the biofilm-embedded bacteria, a method described previously was used [16]. Briefly, the content of each well was scraped and suspended in sterile PBS pH 7.4 (PBS). The total viable counts were determined using serial dilutions and the drop plating method described by Miles, Misra [15] (plating in PCA followed by 24 h incubation), and the results given in log reduction of viable cells, calculated according to the equation below:

Log viable cells reduction = log CFU_positive control_ − log CFU_assay_.

#### 2.5.2. Biofilm Biomass

Total biofilm biomass was determined using the crystal violet method as described by Stepanović, Vuković [17]. Briefly, after the removal of non-adherent cells, the biofilms were fixed with absolute ethanol (Panreac, Barcelona, Spain) for 1 min, stained with crystal violet (1 min) and, after a thorough washing, the content of each well was resuspended in acetic acid (0.1% (*v v*^−1^)) and the OD at 660 nm was read using a microplate reader (Fluostar Optima; BMG Labtech, Ortenberg, Germany). Results were given in biomass formation inhibition percentage according to the following formula:

%biomass formation inhibition= 100 − (OD_assay_/OD_positive control_) × 100.

#### 2.5.3. Biofilm Metabolic Activity

The metabolic activity was assessed using the 2,3-bis(2-methoxy-4-nitro-5-sulfo-phenyl)-2H-tetrazolium—caboxanilide (XTT) colorimetric method as described by Machado, Graça [18]. After a 3 h incubation with 150 mg L^−1^ XTT (Sigma, St. Louis, MO, USA) and 10 mg L^−1^ phenazine methosulfate (PMS, Sigma, St. Louis, MO, USA) at 37 °C, the OD at 485 nm was measured using a Fluostar Optima microplate reader (BMG Labtech, Ortenbeg, Germany). The results were given in metabolic activity inhibition percentage calculated according to the following formula:

%metabolic activity inhibition = 100 − (OD_assay_/OD_positive control_) × 100.

### 2.6. Impact on Bacterial Adhesion—Adhesion to Polystyrene (PS)

The test solutions (specified in Section 2.2) prepared using saline were inoculated at 2% (*v v*^−1^), aliquoted into sterile polystyrene microtiters (Nunc, Darmstadt, Germany) and allowed to incubate for 3 h at 37 °C. After this period, the content of each well was discarded and washed with sterile saline and processed as described above to determine the biofilms’ biomass (Section 2.5.2) [1]. Inoculated saline was used as a positive control and all assays were performed in triplicate. The results were given in percentage of adhesion inhibition according to the equation below.

%adhesion inhibition = 100 − (OD_assay_/OD_positive control_) × 100

### 2.7. Impact on Bacterial Adhesion—Adhesion to Polystyrene Pre-Treated with Rabbit Plasma

Aliquots of rabbit plasma (rp) were used to fill 96-well PS microtiters (Nunc, Darmstadt, Germany) and incubated for 24 h at 37 °C to allow for protein adsorption to the PS surface. After this period, the plasma was discarded and the wells washed with sterile saline solution [1,19]. From this point forward, two different approaches were used. In the first, bacteria were incubated, for 3 h at 37 °C, with the test solutions (PS-rp). In the second, the wells were exposed to the test solutions first (37 °C, 1 h) and only then to the microorganisms (37 °C, 3 h) (PS-rp-ts). Both protocols are fully described elsewhere [1]. Inoculated saline was used as a positive control and all assays were performed in triplicate. The results were given in percentage of adhesion inhibition according to the equation below.

%adhesion inhibition = 100 − (OD_assay_/OD_positive control_) × 100

### 2.8. Statistical Analysis

Statistical analysis of the results was performed using IBM’s SPSS Statistics v21.0.0.0 (New York, NY, USA). The normality of the distributions was assessed using Shapiro–Wilk’s test. When comparing the different test solutions, One-way ANOVA coupled with Tukey’s post hoc test was used. The time inhibition curves were compared using the repeated measures test (to compare the behavior throughout the 24 h period). Differences were considered significant for *p*-values inferior to 0.05.

## 3. Results

### 3.1. Impact upon Staphylococcus Growth

**Figure 1:**
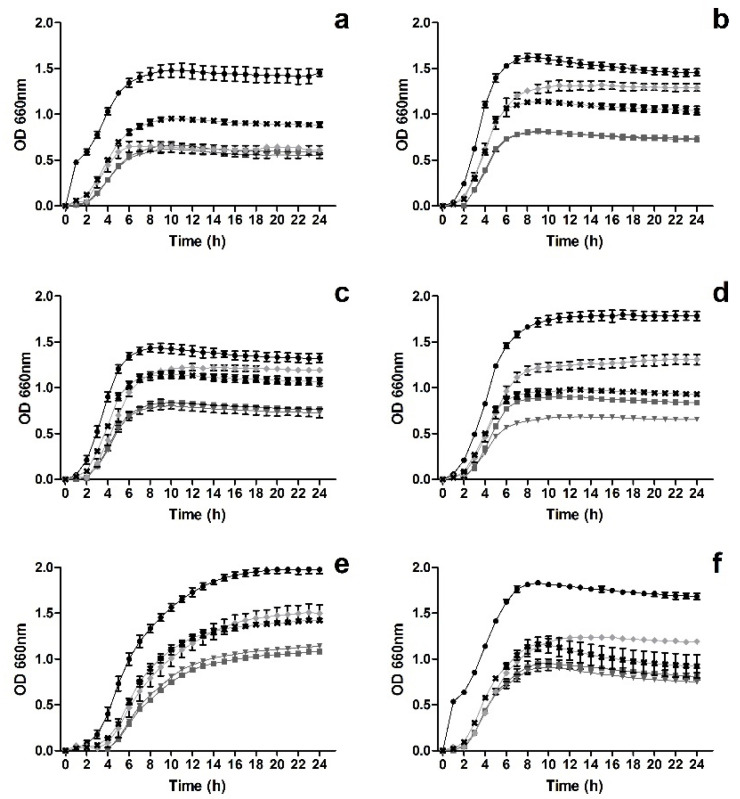
Time inhibition curves drawn for MSSA R (**a**), MSSA CI (**b**), MRSA R (**c**), MRSA CI (**d**), MRSE (**e**) and VRSA (**f**) when exposed to M3Glu at 500 µg mL^−1^ (■), M3Glu at 250 µg mL^−1^ (◆), cM3Glu/NChA (▼ 500 µg mL^−1^ M3Glu with 100 µg mL^−1^ NChA), M3Glu/NChA (✖; 250 µg mL^−1^ M3Glu with 50 µg mL^−1^ NChA) and for the positive control (⬤).

As can be seen in Figure 1, all tested solutions (comprising M3Glu alone or M3Glu mixed with NChA) were capable of reducing the growth of all staphylococci tested. 

For MRSE (Figure 1e), higher concentrations of anthocyanins allowed for higher inhibitions of bacterial growth, with the presence of NChA having no significant (*p* > 0.05) impact upon the results. When considering MSSA R (Figure 1a), no significant (*p* > 0.05) differences were found between the inhibitions observed for the most concentrated M3Glu and NChA mixture (500 µg mL^−1^ M3Glu with 125 µg mL^−1^ NChA, cM3Glu/NChA), M3Glu at 500 µg mL^−1^ and 200 µg mL^−1^, thus indicating that the presence of NChA did not grant any additional inhibition when the anthocyanin was present at 500 µg mL^−1^. When considering the less concentrated M3Glu and NChA mixture (200 µg mL^−1^ M3Glu with 50 µg mL^−1^ NChA, M3Glu/NChA), it can be seen that the inhibition induced by the anthocyanin alone (at 250 µg mL^−1^) was hindered in the presence of NChA, as this combination allowed for higher OD values and an earlier beginning of the log phase. Interestingly, for MSSA CI, MRSA R and VRSA (Figure 1b,c,f), while for the highest concentration of M3Glu (M3Glu at 500 µg mL^−1^ and cM3G/NChA) no significant (*p* > 0.05) differences were observed, the presence of NChA led to an increase in the inhibitory activity when the anthocyanin was present at 250 µg mL^−1^. On the other hand, for MRSA CI (Figure 1d), the presence of NChA in the test solution increased the inhibition observed in comparison to the counterpart solution with M3Glu alone.

**Figure 2:**
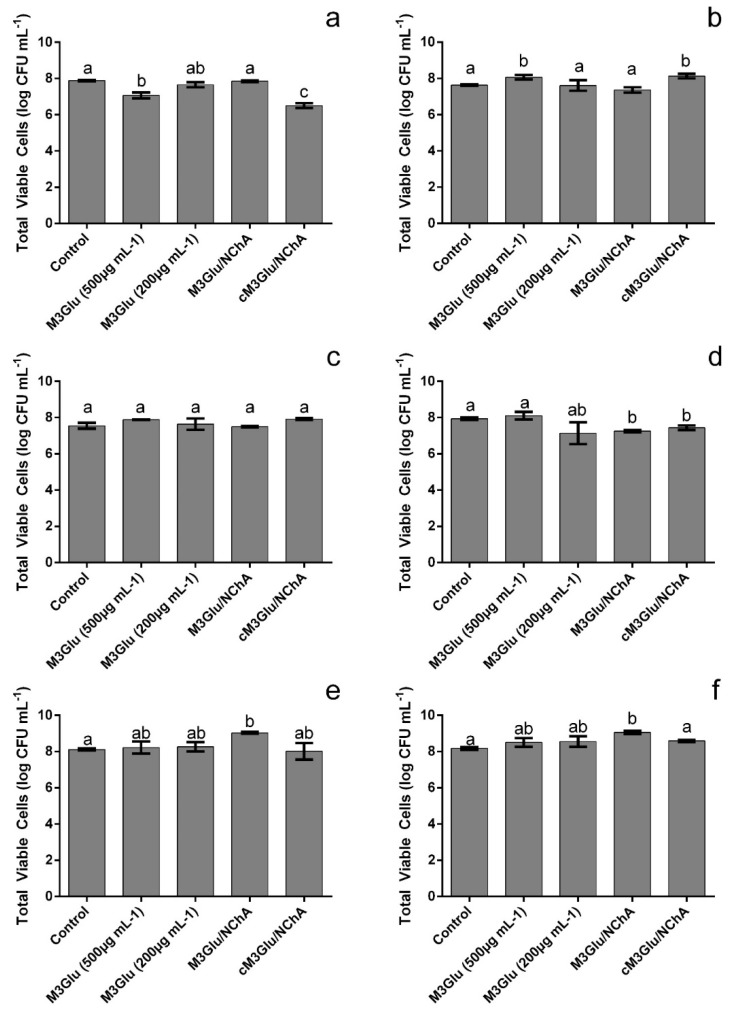
Total viable cells after 24 h exposure of MSSA R (**a**), MSSA CI (**b**), MRSA R (**c**), MRSA CI (**d**), MRSE (**e**) and VRSA (**f**) to the different test solutions. The different letters indicate the statistically significant (*p* < 0.05) differences between the bars.

Despite the overall inhibitions in OD observed (after 24 h) in Figure 1, when contemplating the total viable cells (Figure 2) it can be seen that not all *Staphylococcus* were effectively inhibited by the test solutions. 

For instance, MSSA CI, MRSA R, MRSE and VRSA growth was not significantly (*p* > 0.05) inhibited (Figure 1b,c,e,f). In fact, in some cases, the total viable counts were higher than those registered for the control with the increase ranging from 0.44 log (MSSA CI exposed to 500 µg mL^−1^ of M3Glu) to 0.91 log (MRSE exposed to M3Glu/NChA). Nevertheless, some inhibitions were still observed for MSSA R and MRSA CI, viz., M3Glu (500 µg mL^−1^) and cM3Glu/NChA (500 µg mL^−1^ M3Glu with 100 µg mL^−1^ NChA) were capable of inducing reductions up to 1.4 log of MSSA R viable cells and both mixtures with NChA caused an average of 0.6 log reduction of the total viable cells of MRSA CI.

### 3.2. Impact upon Staphylococcus Biofilms

**Figure 3:**
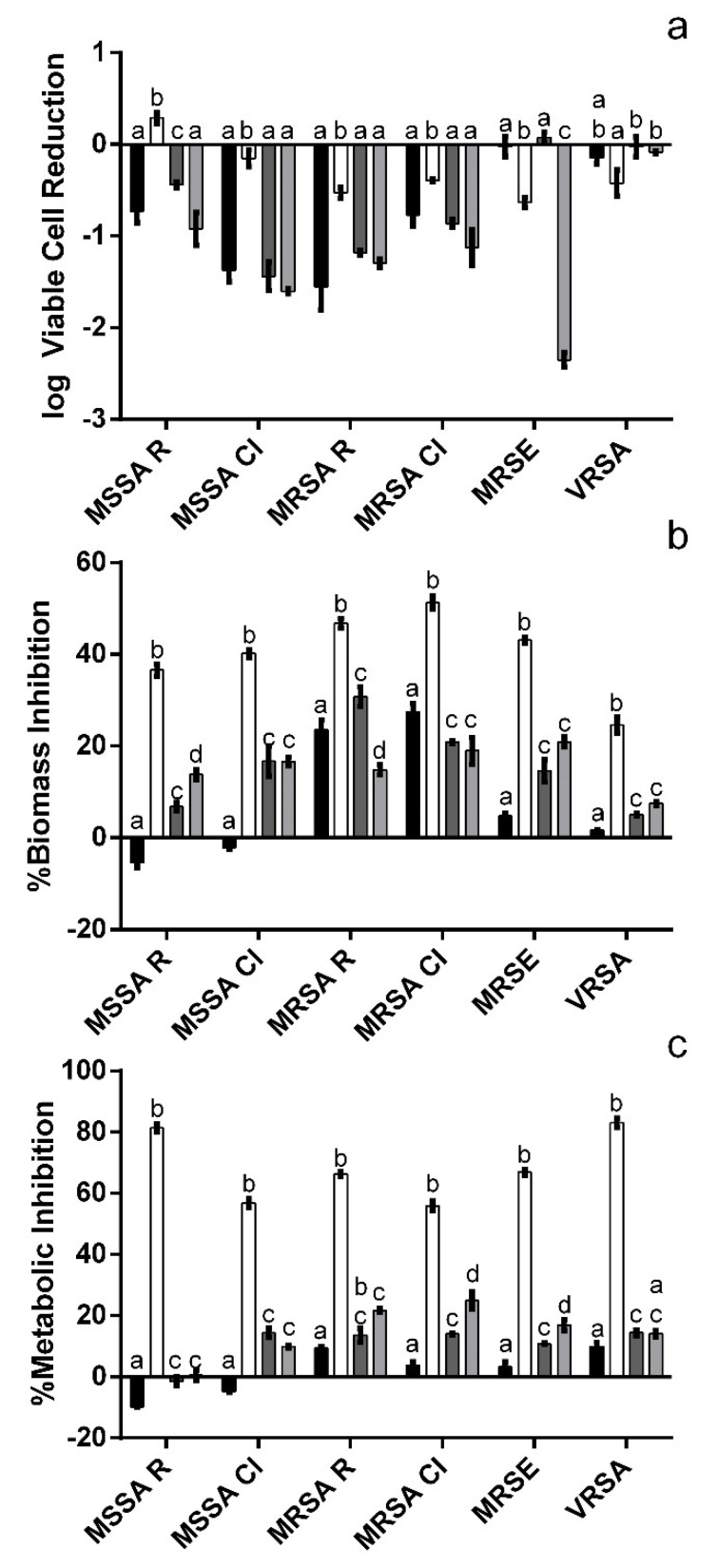
Effect of the test solutions upon biofilms’ viable cells (**a**), biomass (**b**) and metabolic activity (**c**) when exposed to 500 µg mL^−1^ M3Glu (■), 250 µg mL^−1^ M3Glu (□), cM3Glu/NChA (■; 500 µg mL^−1^ M3Glu with 100 µg mL^−1^ NChA) and M3Glu/NCh3 (■; 250 µg mL^−1^ M3Glu with 50 µg mL^−1^ NChA). The letters above each bar indicate the statistically significant (*p* < 0.05) differences found for each microorganism.

As can be seen in Figure 3a, overall, all test solutions were capable of inducing a reduction of the viable, biofilm-entrapped cells though some exceptions were observed. 

Namely, for MSSA R exposed to 250 µg mL^−1^ a small increase in viable cells was observed (0.29 log), while for MRSE (500 µg mL^−1^ M3Glu and cM3Glu/NChA), VRSA (500 µg mL^−1^ M3Glu, cM3Glu/NChA and M3Glu/NChA) and MSSA CI (250 µg mL^−1^ M3Glu) no significant (*p* > 0.05) variations were observed. For MSSA and MRSA (both R and CI strains), as could be expected, a direct relation between concentration and inhibition was observed with the most concentrated M3Glu solution (500 µg mL^−1^) being more effective (by an average of 1.0 log cycles) in reducing the amount of biofilm-entrapped cells than its diluted counterpart (250 µg mL^−1^). However, when considering the M3G solutions with NChA, no statistically significant (*p* > 0.05) differences were found between both solutions regardless of their concentration (cM3Glu/NChA and M3Glu/NChA). The only exception was observed for MSSA R where the most diluted solution was more effective than the concentrated one, an effect also observed for MRSE in the presence of M3Glu and NChA mixed solutions.

Interestingly, when observing the results pertaining to the biofilms’ biomass (Figure 3b) and metabolic activity (Figure 3c), M3Glu at 250 µg mL^−1^ (overall less effective in reducing the biofilm-entrapped cells) produced the highest inhibitions observed. For instance, for MSSA R the 250 µg mL^−1^ solution, while promoting a 0.29 log increase in viable cells, also allowed for a 40% inhibition of total biomass and 81% inhibition of metabolic activity. This scenario is similar to the one observed for all other staphylococci, except VRSA, where the 250 µg mL^−1^ solution is the most effective in reducing the biofilm-entrapped cells.

A higher concentration of anthocyanin (M3Glu at 500 µg mL^−1^) exerted little to no metabolic inhibition upon any of the strains tested (less than 10%) and the same was observed for biomass inhibition (less than 5%) for all bacteria, except MRSA R and MRSA CI (23.6% and 27.4% inhibition, respectively). On another note, the addition of NChA led to significantly (*p* < 0.05) lower inhibition values for the less concentrated mixed solution (M3Glu/NChA). Conversely, when considering cM3Glu/NChA, the addition of NChA caused a significant (*p* < 0.05) increase in the inhibitions observed for biomass (all staphylococci except MRSA CI) and metabolic activity (all bacteria bar VRSA).

### 3.3. Impact upon Staphylococcus Adhesion

**Figure 4:**
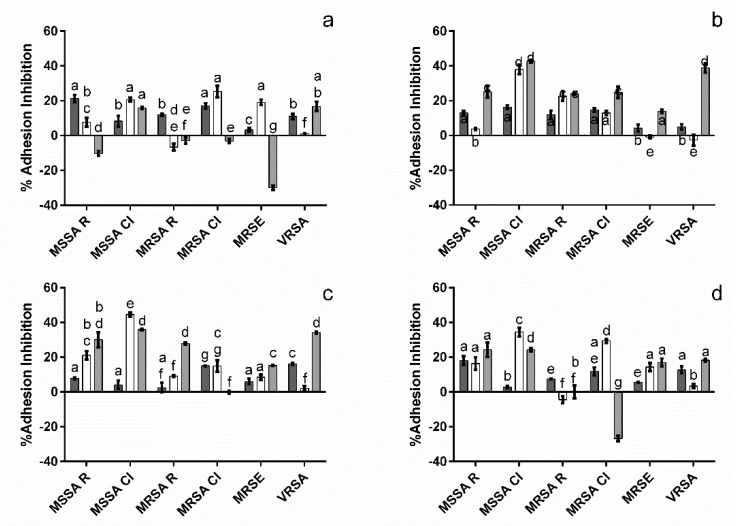
Effect of M3Glu (500 µg mL^−1^ (**a**) and 250 µg mL^−1^ (**b**)), cM3Glu/NChA ((**c**); 500 µg mL^−1^ M3Glu with 100 µg mL^−1^ NChA) and M3Glu/NChA ((**d**); 250 µg mL^−1^ M3Glu with 50 µg mL^−1^ NChA) upon bacterial adhesion to PS (■), PS-rp (□) and PS-rp-ts (■). The different letters indicate the statistically significant (*p* < 0.05) differences between the bars.

To ascertain the anthocyanin and anthocyanin/NChA mixture’s potential to block staphylococcal binding to surfaces, antiadhesion studies were carried out considering both a plain polystyrene (PS) surface and a PS surface coated with plasmatic proteins (PS-rp, to mimic exposure to a cytoplasmatic environment) [19]. All of the tested solutions were capable of inhibiting bacterial adhesion to PS surfaces (Figure 4) with a range of inhibitions from 2.9 (M3Glu/NChA against MSSA CI) to 21.3% (500 µg mL^−1^ M3Glu against MSSA R), with the only exception being MRSA R exposed to cM3Glu/NChA. 

When considering the PS-rp assay, more cases where no inhibition was observed were registered; VRSA for all solutions except M3Glu/NChA, MRSA R for M3Glu (500 µg mL^−1^) or M3Glu/NChA and M3Glu (250 µg mL^−1^) for MRSE. Moreover, it is interesting to note that while more microorganisms appear to be less susceptible to the compounds, the maximum inhibition values are also significantly (*p* < 0.05) higher (ca. 40%), though that did not always translate into higher inhibition values for PS-rp (when compared to PS). 

## 4. Discussion

In an earlier work, it was reported that an anthocyanin-rich blueberry extract (whose main anthocyanin was M3Glu) that contained NChA was capable of fully inhibiting the growth of MSSA R and MRSA R. This activity was observed for 1000 (184.7 µg mL^−1^ total anthocyanins and 32.2 µg mL^−1^ NChA) and 500 (92.35 µg mL^−1^ total anthocyanins and 16.1 µg mL^−1^ NChA) µg mL^−1^ extract [1]. However, in the present work the test solutions, even when at similar or higher concentrations of pure compounds, were unable to exert an inhibition as strong as that of the extract. As it comprised several different compounds and not only the two compounds tested, some of the loss of activity may be explained by their absence. Moreover, it is interesting to note the lack of parity between the inhibitions observed between the total viable count determination after 24 h and the differences in OD observed in the same incubation period. As the spectrophotometric method measures the overall capacity to absorb light and anthocyanins are pigments, some interferences may occur. Furthermore, as the anthocyanins’ chemical nature allows them to shift between forms (with different colors and absorbance spectra) depending on the environmental pH values which, in turn, vary as a result of the bacterial metabolism, this measurement may be somewhat biased [2].

It is interesting to note that, for most *Staphylococcus* strains, the lower anthocyanin concentrations were capable of inducing a reduction in both biomass and metabolic activity, even if this inhibition was not coupled with strong reductions of the biofilm-entrapped cells. This fact indicates that, while the overall amount of cells may not be the lowest, the anthocyanin appears to make them less active and less surrounded by the polymeric matrix that, in turn, helps in the protection of biofilm-embedded bacterial cells [20]. Considering that one of the mechanisms through which biofilms are thought to grant resistance is by obstructing the antimicrobials’ access to the bacterial cells, this reduction in biomass may be particularly interesting to explore in combination with other antibiotics/antimicrobials [20,21]. However, for the higher anthocyanin concentrations tested the effects appear to be the opposite, i.e., there was a higher reduction in viable biofilm-entrapped cells but not as strong reductions of either overall biomass or metabolic activity. This indicates that, while there were fewer viable cells, they had to be more metabolically active and produce more extracellular matrix to compensate for the differences observed. It is possible that the bacteria, perceiving the presence of less favorable environmental conditions, increased their metabolic activity in order to compensate for this stress, namely, through an increase in the production of exopolysaccharide (EPS) [22].

On a different note, the addition of NChA had no ubiquitous effect, as in some cases it promoted the anthocyanin’s effect and, in others, it hindered it. This demonstrated the importance of the overall chemical context when considering the activity of natural plant extracts. Moreover, this accentuates the need to better understand potential synergies or antagonisms that are established between compounds in order to better pinpoint the active principles behind an extract’s biological activity.

Overall, the tested solutions were capable of reducing the biofilms formed by the different staphylococci strains considered and they did so at concentrations that had little to no impact upon the overall bacterial growth. This may be interesting as it allows for a reduction of overall surface colonization without promoting the development of bacterial resistance [23,24,25].

Overall, the tested solutions were capable of inhibiting *Staphylococcus* adhesion to PS. However, when considering the plasma-treated surface (a closer mimic of a biological environment) some adhesion inhibitions were significantly lower while others were significantly higher. This lack of a ubiquitous behavior is unlike what has been observed in a previous work with a blueberry extract (rich in anthocyanins, particularly M3Glu and possessing NChA) where the pre-treatment of PS surfaces with rabbit plasma led to higher inhibition percentages than when adhesion to plain PS was considered [1]. This difference between extract and individual compounds is a possible consequence of the extracts’ complexity as the array of anthocyanins may allow for synergistic activities that M3Glu alone, even when in the presence of NChA, is not able to reproduce.

On another note, to try and ascertain how the interaction of the compounds with the adsorbed proteins could affect the adhesion process, a third surface, sequentially exposed to rabbit plasma, test solutions and microorganisms, was considered (PS-rp-ts). Overall, four different behaviors were observed: (i) no significant differences (*p* > 0.05) were found between the adhesion to PS-rp and PS-rp-ts surfaces, indicating that the inhibitory effect observed is a consequence of the interactions between M3Glu, NChA and the adsorbed proteins [26]. (ii) Adhesion inhibition was higher for PS-rp than for PS-rp-ts, however, the presence of inhibition in the latter indicates that at least part of the inhibition observed was mediated by interactions between the compounds and the adsorbed proteins. (iii) Inhibition of adhesion was lower for PS-rp than for PS-rp-ts. This occurs only for VRSA, which may indicate that the compounds are less effective in blocking bacterial adhesion when in the presence of VRSA, either because the bacteria are faster to adhere than the compounds are to interact or because VRSA interacted with the compound, reducing its effectiveness. (iv) PS-rp inhibition values are higher than those observed for PS-rp-ts and the latter are negative, thus signaling that the interaction of the compounds with the adsorbed proteins may accentuate bacterial adhesion.

Overall, when considering the activity of the isolated compounds it is significantly lower than that observed for a similar extract. Considering the lack of evidence on the antimicrobial activity of pure anthocyanins and the lack, to the best of our knowledge, of mechanistic papers, we can only hypothesize why this occurs. The addition of NChA proved to cause a significant variation in M3Glu activity, thus demonstrating that one of the possible explanations for the differences is the absence of synergies with other compounds. Another reason that may explain these differences, particularly when considering the effect of the addition of an acid, is the environmental pH values. In solution, anthocyanins may be found in several different forms depending on the environmental pH; at low pH, the main form is the flavylium cation which is positively charged while at the pH in which these inhibitions were observed (ca. 6.5—data not shown) the major form is the neutral quinoidal base [27]. Bacterial cells are electronegative entities which makes them more prone to interact with positively charged compounds, thus the environmental pH values may explain the comparatively lower activity observed as well as prove to be an important subject to be studied in the future [28].

## 5. Conclusions

In conclusion, while not effective in inhibiting the amount of planktonic staphylococci after 24 h, M3G (alone and in the presence of NChA) was effective in inhibiting biofilm formation and *Staphylococcus* adherence. This capacity to reduce adhesion and biofilms without reducing bacterial growth is an important characteristic as it reduces the likeliness of resistance development, making M3Glu an interesting alternative antimicrobial compound. Furthermore, their capacity to reduce bacterial adhesion makes them interesting additives to be used when bacterial infection/colonization is an important factor to control, though further studies seeking to understand the role of environmental pH may be of importance as it may allow for better inhibitions. However, due to the intrinsic cost of attaining pure standards, they may not be ready for industrial application except in cases where the final product exhibits high commercial value. An example could be in the development of topical treatments for individuals with eczema, psoriasis or other skin diseases that can be correlated with a hegemony of *Staphylococcus* populations.

## Data Availability

The data presented in this study are available in the article.

## References

[1] Silva S., Costa E., Mendes M., Morais R., Calhau C., Pintado M. (2016). Antimicrobial, antiadhesive and antibiofilm activity of an ethanolic, anthocyanin-rich blueberry extract purified by solid phase extraction. J. Appl. Microbiol..

[2] Castañeda-Ovando A., Pacheco-Hernandez M.D.L., Páez-Hernández M.E., Rodríguez J.A., Galán-Vidal C.A. (2009). Chemical studies of anthocyanins: A review. Food Chem..

[3] Silva S., Costa E.M., Calhau C., Morais R.M., Pintado M.E. (2015). Anthocyanin extraction from plant tissues: A review. Crit. Rev. Food Sci. Nutr..

[4] Cisowska A., Wojnicz D., Hendrich A.B. (2011). Anthocyanins as Antimicrobial Agents of Natural Plant Origin. Nat. Prod. Commun..

[5] Badshah H., Kim T.H., Kim M.O. (2015). Protective effects of Anthocyanins against Amyloid beta-induced neurotoxicity in vivo and in vitro. Neurochem. Int..

[6] Wallace T.C., Giusti M.M. (2013). Anthocyanins in Health and Disease.

[7] Zhang Y., Lin Y., Huang L., Tekliye M., Rasheed H.A., Dong M. (2020). Composition, antioxidant, and anti-biofilm activity of anthocyanin-rich aqueous extract from purple highland barley bran. LWT.

[8] Correia P., Araújo P., Ribeiro C., Oliveira H., Pereira A., Mateus N., de Freitas V., Brás N., Gameiro P., Coelho P. (2021). Anthocyanin-Related Pigments: Natural Allies for Skin Health Maintenance and Protection. Antioxidants.

[9] Puupponen-Pimiä R., Nohynek L., Meier C., Kähkönen M., Heinonen M., Hopia A., Oksman-Caldentey K.-M. (2001). Antimicrobial properties of phenolic compounds from berries. J. Appl. Microbiol..

[10] Chambers H.F. (2001). The changing epidemiology of *Staphylococcus aureus*?. Emerg. Infect. Dis..

[11] Livermore D.M. (2000). Antibiotic resistance in staphylococci. Int. J. Antimicrob. Agents.

[12] Carbon C. (2000). MRSA and MRSE: Is there an answer?. Clin. Microbiol. Infect..

[13] Carvalho F.B., Gutierres J.M., Bohnert C., Zago A.M., Abdalla F.H., Vieira J.M., Palma H.E., Oliveira S.M., Spanevello R.M., Duarte M.M. (2015). Anthocyanins suppress the secretion of proinflammatory mediators and oxidative stress, and restore ion pump activities in demyelination. J. Nutr. Biochem..

[14] (2009). M07-08–Methods for Dilution Antimicrobial Susceptibility Tests for Bacteria that Grow Aerobically.

[15] Miles A.A., Misra S.S., Irwin J.O. (1938). The estimation of the bactericidal power of the blood. Epidemiol. Infect..

[16] Silva S., Costa E.M., Costa M.R., Pereira M.F., Pereira J.O., Soares J.C., Pintado M.M. (2015). Aqueous extracts of Vaccinium corymbosum as inhibitors of *Staphylococcus aureus*. Food Control.

[17] Stepanović S., Vuković D., Dakić I., Savić B., Švabić-Vlahović M. (2000). A modified microtiter-plate test for quantification of staphylococcal biofilm formation. J. Microbiol. Methods.

[18] Machado I., Graça J., Lopes H., Lopes S., Pereira M.O. (2013). Antimicrobial Pressure of Ciprofloxacin and Gentamicin on Biofilm Development by an Endoscope-Isolated *Pseudomonas aeruginosa*. ISRN Biotechnol..

[19] van Loosdrecht M.C.M., Norde W., Lyklema J., Zehnder A.J.B. (1990). Hydrophobic and electrostatic parameters in bacterial adhesion. Aquat. Sci..

[20] Chaignon P., Sadovskaya I., Ragunah C., Ramasubbu N., Kaplan J.B., Jabbouri S. (2007). Susceptibility of staphylococcal biofilms to enzymatic treatments depends on their chemical composition. Appl. Microbiol. Biotechnol..

[21] Fux C.A., Costerton J.W., Stewart P.S., Stoodley P. (2005). Survival strategies of infectious biofilms. Trends Microbiol..

[22] Landini P. (2009). Cross-talk mechanisms in biofilm formation and responses to environmental and physiological stress in *Escherichia coli*. Res. Microbiol..

[23] Costerton J.W., Stewart P.S., Greenberg E.P. (1999). Bacterial Biofilms: A Common Cause of Persistent Infections. Science.

[24] Donlan R.M. (2001). Biofilm Formation: A Clinically Relevant Microbiological Process. Clin. Infect. Dis..

[25] Zimmer K.R., Blum-Silva C.H., Souza A.L.K., Wulffschuch M., Reginatto F.H., Pereira C.M.P., Macedo A.J., Lencina C.L. (2014). The Antibiofilm Effect of Blueberry Fruit Cultivars Against *Staphylococcus epidermidis* and *Pseudomonas aeruginosa*. J. Med. Food.

[26] Soares S., Mateus N., de Freitas V. (2007). Interaction of Different Polyphenols with Bovine Serum Albumin (BSA) and Human Salivary α-Amylase (HSA) by Fluorescence Quenching. J. Agric. Food Chem..

[27] Lopes T.J., Yaginuma S.R., Quadri M.G.N., Quadri M.B. (2011). Evaluation of red cabbage anthocyanins after partial purification on clay. Braz. Arch. Biol. Technol..

[28] Costa E.M., Silva S., Pina C., Tavaria F.K., Pintado M.M. (2012). Evaluation and insights into chitosan antimicrobial activity against anaerobic oral pathogens. Anaerobe.

